# Combined effect of adiposity and elevated inflammation on incident type 2 diabetes: a prospective cohort study

**DOI:** 10.1186/s12933-023-02067-0

**Published:** 2023-12-20

**Authors:** Dan Wu, Yulong Lan, Shuohua Chen, Xiong Ding, Guanzhi Chen, Chutao Wu, Lois Balmer, Wencan Xu, Shouling Wu, Wei Wang

**Affiliations:** 1 Centre for Precision Health, Edith Cowan University School of Medical and Health Sciences, Room 521, Building 21/270 Joondalup Drive, Perth, WA 6027 Australia; 2 Department of Pediatrics, Second Affiliated Hospital of Shantou University Medical College, Shantou, Guangdong China; 3 Department of Endocrinology, The First Affiliated Hospital of Shantou University Medical College, No. 57, Changping Road, Jinping District, Shantou, 515041 Guangdong China; 4 Department of Cardiology, Second Affiliated Hospital of Shantou University Medical College, Shantou, Guangdong China; 5 Department of Cardiology, Kailuan General Hospital, Xinghua East Road, Tangshan, 063000 Hebei China; 6 School of Public Health, Wuhan University, Wuhan, China; 7 China Medical University, Shenyang, China; 8 Department of Emergency, Shantou Central Hospital, Shantou, Guangdong China; 9 Clinical Research Centre, The First Affiliated Hospital of Shantou University Medical College, Shantou, Guangdong China; 10 Beijing Key Laboratory of Clinical Epidemiology, School of Public Health, Capital Medical University, Beijing, China; 11 School of Public Health, Shandong First Medical University & Shandong Academy of Medical Sciences, Tai’an, Shandong China

**Keywords:** Adiposity, Inflammation, Type 2 diabetes, BMI, Longitudinal cohort study

## Abstract

**Background:**

Adiposity and elevated inflammation are two hallmarks of hyperglycemia. However, it is unknown whether clustering of elevated inflammation and adiposity interact act on diabetogenesis and lead to a greater risk for incident type 2 diabetes (T2D).

**Methods:**

Adiposity was indicated by body mass index, waist circumference and ultrasonography-measured fatty liver degrees. Elevated inflammation was indicated as high-sensitivity C-reactive protein levels ≥ 2 mg/L. Time-to-event survival analyses were conducted to investigate the joint effect of adiposity and inflammation on incident T2D on both multiplicative and additive scales.

**Results:**

Among 82,172 non-diabetic participants from a prospective cohort in China, 14,278 T2D occurred over a median follow-up of 11 years. In the multivariable-adjusted model, elevated inflammation [1.12 (1.08‒1.16)] and adiposity [1.76 (1.69‒1.83) for overweight/obesity, 1.49 (1.44‒1.55) for central obesity, and 2.02 (1.95‒2.09) for fatty liver] were significantly associated with incident diabetes. Higher adiposity-associated risks and incidence rates of diabetes were observed with elevated inflammation. When studying the joint effect, the adjusted HRs were 1.77 (1.69‒1.85) for overweight/obesity, 1.14 (1.06‒1.23) for elevated inflammation, and 2.08 (1.97‒2.19) for their joint effect, with a relative excess risk due to interaction of 0.17 (0.05‒0.28). The attributable proportions were 71.30% for overweight/obesity, 12.96% for elevated inflammation, and 15.74% for their interaction. Similar results were observed when adiposity was assessed as waist circumference or fatty liver.

**Conclusions:**

Adiposity and elevated inflammation synergically lead to greater risks of incident diabetes than addition of each individual exposure. Strategies simultaneously targeting both risks should produce more benefits for diabetes prevention than through initiatives directed at each separate risk.

**Supplementary Information:**

The online version contains supplementary material available at 10.1186/s12933-023-02067-0.

## Background

Deeply involving both overnutrition-derived metabolic disorders and elevated inflammation, type 2 diabetes (T2D) has become a global pandemic and poses great health and economic burden to the worldwide population [1]. China was reported to have the largest number of patients with diabetes and spending the second highest amount on diabetes and its complications worldwide [2, 3], with an estimated expenditure of USD 109.0 billion in 2019 [3]. Prevention and control of T2D is a public health priority worldwide, especially in the Chinese population.

The parallel epidemics of obesity and diabetes in past decades have documented a proven role of increased body weight in diabetes prevalence [4]. Most individuals with diabetes were overweight/obese. However, only approximately one-third of obese, insulin-resistant individuals actually develop chronic hyperglycemia and T2D [5]. Apart from genetic predisposition and environmental factors that may account for the risk heterogeneities, chronic inflammation potentially constitutes an important link between obesity and its pathophysiological sequelae [6]. Accumulating data have suggested a pathological role of inflammation in diabetogenesis [7–10]. However, the inflammation-diabetes association is not conclusive. Some studies indicated that the association was mainly affected by increased body weight or liver function [11–14], whereas some studies suggested that a positive association exists in which inflammation is a significant risk factor for diabetes independent of body excess weight [10, 15, 16]. Moreover, conflicting evidence exists for the inflammation-diabetes association in individuals with overweight and obesity. In a nationwide cohort study (CHARLS) in China, the high-sensitivity C-reactive protein (hsCRP, a widely used inflammatory biomarker) association was more prominent in overweight/obese individuals than in those with normal weight/underweight [10]. In contrast, in the Jackson Heart study [11], the hsCRP-associated diabetic risks were not significant in obese African Americans. Despite the conflicting findings, it is still unknown whether subclinical inflammation is merely a marker of T2D or interacts with adiposity and acts on diabetogenesis.

To our knowledge, epidemiological studies considering the potential biological interaction effect of elevated inflammation and adiposity phenotypes on incident T2D are sparse thus far. Therefore, we conducted an analysis based on data from a large-scale, real-life cohort (Kailuan study) to test the hypothesis that elevated inflammation modifies the risks of developing diabetes upon excess weight and thereafter quantitatively calculate the proportions of this joint association for elevated inflammation, adiposity and their interaction.

## Methods

### Study population

We used data from a prospectively designed, real-world, community-based cohort study in China (Kailuan study). The Kailuan study (Trial Registration Number: ChiCTR-TNC-11001489) began in 2006 when 101,510 employees and retirees of the Kailuan community were recruited to participate in a baseline and a biennial follow-up heath examination circle. All medical and lifestyle information, anthropometrics and biological tests were updated every two years. For the current analysis, we excluded those with incomplete data or abnormal values on sociodemographic, weight, height, waist circumference, fatty liver degrees, fasting blood glucose (FBG), triglyceride (TG), high-density lipoprotein (HDL-C) or hsCRP parameters (n = 4,098), those with pre-existing diabetes (n = 8460) or missing follow-up visits from 2006/2007 through December 31, 2020 (n = 6780). A total of 82,172 participants remained eligible for the current analysis (Additional file 1: Figure S1). Our study was approved by the Ethics Committee of Kailuan General Hospital (approval number: 2006–05) and the Human Research Ethics Committee of Edith Cowan University (approval number: 2021–03159-BALMER). All participants agreed to participate in the study and provided informed written consent.

### Exposures

Adiposity was assessed by body mass index (BMI, calculated as measured weight (kg) divided by height in meters square), waist circumference and fatty liver degrees. For BMI-indicated adiposity, underweight was defined as BMI less than 18.5; normal weight, 18.5 to 23.9; overweight, 24 to 27.9, and obesity, 28 or higher, according to the Chinese national standard [17]. Central obesity was defined as a waist circumference of 90 cm or greater for men and 85 cm or greater for women [18]. The severity of fatty liver was differentiated by ultrasonography: mild (diffuse increase in fine echoes in liver parenchyma), moderate (diffuse increase in fine echoes with impaired visualization of the intrahepatic vessel borders and diaphragm), and severe (diffuse increase in fine echoes with nonvisualization of the intrahepatic vessel borders and diaphragm) [19]. Elevated inflammation was defined by a hsCRP value ≥ 2 mg/L [20] or ≥ 3 mg/L [21].

### Outcome

Primary outcomes were prevalence of T2D according to American Diabetes Association criteria [22]. T2D was defined as participants with self-reported diabetes diagnosed by a health professional or self-reported use of oral antidiabetics or with a fasting plasma glucose level of 126 mg/dL or greater. Participants contributed person-time from baseline until the date of diagnosis of diabetes, death, or the last available follow-up visit prior to December 31, 2020, whichever came first.

### Assessment of other confounders

Information on potential confounders (including sociodemographics, anthropometrics, lifestyle factors, family history of diabetes, history of medication use and medical diseases) was collected according to baseline information. Smoking habits were categorized as never, former, or current smoker, and drinking status was grouped as “yes or no”, according to cigarette or alcohol consumption in the past year, as detailed previously [23].

### Statistical analyses

Baseline information was displayed overall and across inflammation levels. The data on covariates were > 99% complete. We used multiple imputation by chained equation techniques to account for missing data under the missing-at-random assumption [24] (missing data are specified in Additional file 1: Table S1). Baseline characteristics were described as the mean with standard deviation (SD), median together with IQR, or numbers and percentages (%), when appropriate. Differences in baseline characteristics between hsCRP < 2 and hsCRP ≥ 2 mg/L were compared using the chi-square test for categorical variables and an unpaired Student’s *t* test or Mann‒Whitney *U* test for continuous variables.

Unadjusted incidence rates (per 1000 person-years) and Kaplan‒Meier failure functions were used to present the absolute risk of T2D. Cox proportional hazards models were used to model time to event for estimation of relative risks of incident T2D upon increased adiposity and inflammation, as alone or jointly, unadjusted and adjusted for potential confounding variables. The multivariable-adjusted models were as follows: Model 1, adjusted for age, sex, education, smoking and drinking status, physical activities, family history of diabetes, antihypertensives and lipid-lowering drugs; Model 2, further adjusted for systolic blood pressure (continuous), *log*(TG/HDL-C) (continuous); and Model 3, additionally adjusted for log(hsCRP) or BMI levels for each isolated exposure. Multivariable adjusted models for adiposity components (BMI-indicated overweight, central obesity and fatty liver statuses) were then repeated in two different levels of inflammation (elevated inflammation or not). Likelihood ratio tests evaluated the multiplicative interaction (INTm) between increased adiposity and hsCRP levels in the fully multivariable-adjusted Cox models. The relative excess risk due to interaction (RERI) and attributable proportion due to interaction (AP) were assessed as an index of additive interaction (INTa) [25, 26] between elevated inflammation and adiposity in developing diabetes, with both the absence of elevated inflammation and adiposity as the baseline. Briefly, on the hazard ratio scale, we decomposed the joint excess relative risk for both exposures (HR11-1) into the excess relative risk for elevated inflammation (HR01-1), adiposity (HR10-1), and RERI. Specifically, we have HR11 − 1 = (HR01-1) + (HR10-1) + RERI [27]. We thereafter examined the decomposition of the joint effect: the proportion attributable to elevated inflammation alone (HR01 − 1)/(HR11 − 1), adiposity alone (HR10 − 1)/(HR11 − 1), and the additive interaction RERI/(HR11-1) [26].

To assess the robustness of the findings, sensitivity analyses were performed by excluding T2D onset within the first follow-up survey, excluding participants with known cardiovascular diseases (CVD), excluding those with a hsCRP level ≥ 10 mg/L, or excluding participants with incomplete data.

All statistical analyses were performed with SAS software (version 9.4; SAS Institute, Cary, NC). A two-tailed *P* value < 0.05 was considered statistically significant, except for interaction testing, where a *P* value < 0.1 was considered significant. RERI and AP greater than zero with the 95% CIs did not contain zero and synergic index (S) greater than one with the 95% CIs did not contain one indicate a statistically significant additive interaction.

## Results

Table 1 shows the characteristics of the study population overall and across hsCRP levels. The study participants had a mean (SD) age of 50.4 (12.0) years and a male skewness [66,047 males (80.4%)]. Those with elevated inflammation were more likely to be older, former and current smokers, and prone to have moderate physical activity. A positive correlation was observed between levels of hsCRP and BMI, waist circumference, TGs and blood pressure. Individuals with elevated inflammation tended to have a higher prevalence of adiposity, CVD and medication use of anti-hypertensives and lipid-lowering drugs.

**Table 1:** Baseline characteristics of the study participants

	Total (n = 82,172)	HsCRP < 2 mg/L (n = 60,846)	HsCRP ≥ 2 mg/L (n = 21,326)	*P-*difference
Age, mean (SD), years	50.4 ± 12.0	49.4 ± 11.8	53.2 ± 12.2	< 0.0001
Male, No. (%)	66,047 (80.4)	49,241 (80.9)	16,806 (78.8)	< 0.0001
BMI, mean (SD), kg/m^2^	25.0 ± 3.4	24.7 ± 3.3	25.6 ± 3.7	< 0.0001
BMI-categories, No. (%)				< 0.0001
Underweight/normal weight	33,220 (40.4)	25,937 (42.6)	7283 (34.1)	
Overweight	34,273 (41.7)	25,283 (41.6)	8990 (42.2)	
Obese	14,679 (17.9)	9626 (15.8)	5053 (23.7)	
Waist circumference, mean (SD), cm	86.6 ± 9.7	85.6 ± 9.4	89.3 ± 10.1	< 0.0001
Waist circumference-categories, No. (%)				< 0.0001
Non abdominal obesity	48,935 (59.6)	38,859 (63.9)	10,076 (47.3)	
Abdominal obesity	33,237 (40.4)	21,987 (36.1)	11,250 (52.8)	
Fatty liver degrees, No. (%)				< 0.0001
No fatty liver	57,611 (70.1)	44,386 (73.0)	13,225 (62.0)	
Gentle fatty liver	16,548 (20.1)	11,378 (18.7)	5170 (24.2)	
Moderate and severe fatty liver	8013 (9.8)	5082 (8.3)	2931 (13.7)	
hsCRP, median (IQR), mg/L	0.8 (0.3–2.1)	0.5 (0.2–0.9)	4.5 (2.8–7.8)	< 0.0001
SBP, mean (SD), mm Hg	129.7 ± 20.4	128.7 ± 20.0	132.4 ± 21.5	< 0.0001
DBP, median (IQR), mm Hg	80.0 (78.0–90.0)	80.0 (76.7–90.0)	80.7 (79.3–90.0)	< 0.0001
HDL-C, median (IQR), mmol/L	1.50 (1.28–1.76)	1.51 (1.28–1.77)	1.49 (1.27–1.75)	0.1001
TC, mean (SD), mmol/L	4.9 ± 1.1	4.9 ± 1.1	4.9 ± 1.1	0.0024
TG, median (IQR), mmol/L	1.25 (0.88–1.88)	1.22 (0.87–1.83)	1.31(0.92–2.00)	< 0.0001
Family history of diabetes, No. (%)	6823 (8.3)	5230 (8.6)	1593 (7.5)	< 0.0001
Education, No. (%)				< 0.0001
Less than high school	64,993 (79.1)	47,916 (78.8)	17,077 (80.1)	
High school and above	17,179 (20.9)	12,930 (21.2)	4249 (19.9)	
Current drinker, No. (%)	31,327 (38.1)	24,134 (39.7)	7193 (33.7)	< 0.0001
Smoking habits, No. (%)				< 0.0001
Never smoker	49,046 (59.7)	37,210 (61.2)	11,836 (55.6)	
Ever smoker	4457 (5.4)	3170 (5.2)	1287 (6.0)	
Current smoker	28,669 (34.9)	20,466 (33.6)	8203 (38.4)	
Physical activities, No. (%)				< 0.0001
Low	7433 (9.0)	5687 (9.4)	1746 (8.2)	
Moderate	62,122 (75.6)	45,794 (75.2)	16,328 (76.5)	
High	12,617 (15.4)	9365 (15.4)	3252 (15.3)	
CVD, No. (%)	2458 (3.0)	1588 (2.6)	870 (4.1)	< 0.0001
Medication use, No. (%)				
Antihypertensives	2238 (2.7)	1426 (2.3)	812 (3.8)	< 0.0001
Statin	175 (0.2)	117 (0.2)	58 (0.3)	< 0.0001
Fibrate	64 (0.08)	38 (0.06)	26 (0.12)	0.0299

*BMI* body mass index, *CVD* cardiovascular diseases, *HDL-C* high-density lipoprotein cholesterol, *hsCRP* high-sensitivity C-reactive protein, *SBP* systolic blood pressure *TC* total cholesterol, *TG* triglyceride

**Fig. 1:**
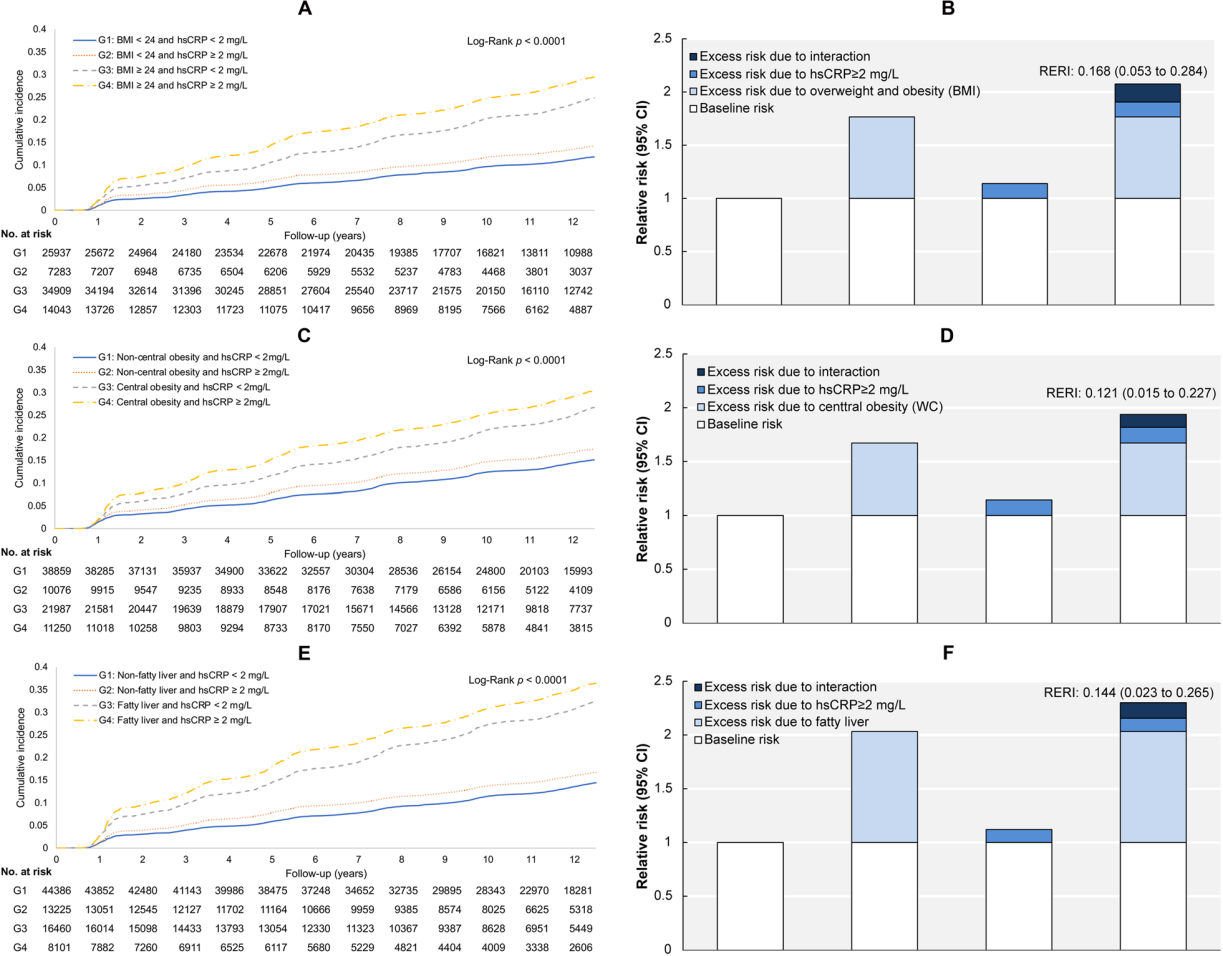
Cumulative incidence of type 2 diabetes across co-exposure to elevated inflammation and adiposity and their relative risks of contributions to incident type 2 diabetes. For the analysis of the supra-additive effect, the baseline category was defined as having no exposure to adiposity or elevated inflammation. **A** and **B** display the results when adiposity was indicated by BMI. **C** and **D** display the results when adiposity was indicated by waist circumference. **E** and **F** display the results when adiposity was indicated by fatty liver status. In the RERI analysis, all models were adjusted for age, sex, education, smoking status, drinking status, physical activity, family history of diabetes, anti-hypertensives, lipid-lowering drugs, *log*(TG/HDL-C) (continuous), and SBP (continuous). RERI, relative excess risk due to interaction; others as Table 1

During a median follow-up of 10.9 (IQR: 6.8–12.6) years, 14,278 T2D cases were recorded among 82,172 non-diabetic participants. We observed positive associations between isolated exposure to elevated inflammation or adiposity and the risk of incident T2D after adjusting for potential confounders, including sociodemographic, lifestyle factors, family history of diabetes, medication use, blood pressure and lipid profiles. Compared to hsCRP < 2 mg/L, hsCRP ≥ 2 mg/L had an adjusted risk of 1.20 (95% CI: 1.16‒1.25), with each 1-SD increase in log-normalized hsCRP associated with a risk of 1.12 (95% CI: 1.10‒1.14) (*P*-trend: < 0.0001). Further adjusting for BMI (continuous) attenuated the hsCRP-diabetes association; however, it remained significant (Additional file 1: Table S2). For adiposity (Table 2), in the overall population, the risks of incident diabetes were 1.76 (95% CI: 1.69‒1.83) for overweight/obesity (vs BMI < 24 kg/m^2^), 1.49 (95% CI: 1.44‒1.55) for central obesity (vs non-central obesity), and 2.02 (95% CI: 1.95‒2.09) for fatty liver (vs non-fatty liver) in the multivariable adjusted model. The adiposity-associated T2D incidence rates and risks appeared to be greater in those with elevated inflammation. In the hsCRP < 2 mg/L stratum, compared to those without adiposity, the risks of incident diabetes were 1.75 (95% CI: 1.67‒1.84) for overweight/obesity, 1.49 (95% CI: 1.43‒1.55) for central obesity, and 2.01 (95% CI: 1.93‒2.10) for fatty liver. In contrast, in the hsCRP ≥ 2 mg/L stratum, the risks of incident diabetes were 1.85 (95% CI 1.71‒1.99) for overweight/obesity, 1.55 (95% CI 1.45‒1.65) for central obesity, and 2.07 (95% CI 1.94‒2.20) for fatty liver. This trend persisted in the associations between waist circumference or fatty liver and incident diabetes after further adjusting for BMI. We additionally studied the BMI-diabetes and fatty liver-diabetes associations by regrouping BMI as underweight/normal weight, overweight, and obesity and regrouping fatty liver degrees as non-fatty liver, mild, moderate and severe fatty liver and yielded similar results regarding the higher diabetic risks and incidence rates in the subgroups (Additional file 1: Tables S3–4). Tests for multiplicative interactions were not significant when these variables were tested as the study categories. The results with product terms between adiposity categories and inflammation subgroups were similar to the primary findings (Additional file 1: Table S5). We additionally address the biological interactions by assessing the INTa. There were significant additive interactions between adiposity and elevated inflammation in developing diabetes (*P* < 0.001). In the fully multivariable-adjusted model, the adjusted HRs for T2D were 1.77 (95% CI 1.69‒1.85) for overweight and obesity status, 1.14 (95% CI 1.06‒1.23) for elevated inflammation, and 2.08 (95% CI 1.97‒2.19) for their joint effect, with an RERI of 0.17 (95% CI: 0.05‒0.28). All the AP and S are also statistically significant for indicating an additive interaction. The attributable proportions of the joint effect were 71.30% for overweight/obesity, 12.96% for elevated inflammation, and 15.74% for their interaction. Likewise, we documented similar results regarding RERI and attributable proportions in central obesity concurrent with elevated inflammation in developing diabetes. Fatty liver tended to have higher diabetic risks compared to the other 2 study adiposity indexes. The attributable proportions were 79.23% for fatty liver, 9.23% for elevated inflammation, and 11.54% for their interaction (Table 3). When we examined the association of joint categories of adiposity and elevated inflammation across different inflammation levels, each study component of adiposity was consistently associated with a higher risk and incidence rates of T2D (Fig. 1; Additional file 1: Tables S6–S8). Compared with the reference group (those with no exposure to elevated inflammation and adiposity), those with elevated inflammation concomitant with adiposity had the highest diabetic risks [2.08 (95% CI: 2.97‒2.19) for overweight/obesity, 1.94 (95% CI: 1.85‒2.03) for central obesity, 2.30 (95% CI: 2.19‒2.42) for fatty liver].

**Table 2:** Adiposity-associated risks of incident type 2 diabetes in the entire population and across different hsCRP levels

	BMI, HRs (95% CIs)		Waist circumference, HRs (95% CIs)		Fatty liver, HRs (95% CIs)		*P*-trend
	BMI < 24 kg/m^2^	BMI ≥ 24 kg/m^2^	Non-central obesity	Central obesity	Non-fatty liver	Fatty liver	
Entire population							
Event/total	3443/33220	10,835/48952	6446/48935	7832/33237	7169/57611	7109/24561	
Incidence rates	10.37	23.82	13.34	25.79	12.60	32.64	
Unadjusted model	Reference	2.29 (2.20‒2.38)	Reference	1.93 (1.87‒1.99)	Reference	2.58 (2.50‒2.67)	< 0.0001
Model 1	Reference	2.21 (2.12‒2.29)	Reference	1.82 (1.76‒1.89)	Reference	2.50 (2.42‒2.58)	< 0.0001
Model 2	Reference	1.76 (1.69‒1.83)	Reference	1.49 (1.44‒1.55)	Reference	2.02 (1.95‒2.09)	< 0.0001
Model 3	-	-	Reference	1.16 (1.11‒1.20)	Reference	1.71 (1.64‒1.77)	< 0.0001
hsCRP < 2 mg/L (9854/60846)							
Event/Total	2573/25937	7281/34909	4947/38859	4907/21987	5307/44386	4547/16460	
Incidence rate	9.87	22.17	12.84	24.09	12.03	30.77	
Unadjusted model	Reference	2.24 (2.14‒2.34)	Reference	1.88 (1.80‒1.95)	Reference	2.55 (2.45‒2.66)	< 0.0001
Model 1	Reference	2.15 (2.05‒2.25)	Reference	1.77 (1.70‒1.85)	Reference	2.46 (2.37‒2.56)	< 0.0001
Model 2	Reference	1.75 (1.67‒1.84)	Reference	1.49 (1.43‒1.55)	Reference	2.01 (1.93‒2.10)	< 0.0001
Model 3	–	–	Reference	1.14 (1.09‒1.19)	Reference	1.68 (1.61‒1.76)	< 0.0001
hsCRP ≥ 2 mg/L (4424/21326)							
Event/Total	870/7283	3554/14043	1499/10076	2925/11250	1862/13225	2562/8101	
Incidence rate	12.21	28.08	15.32	29.26	14.56	36.61	
Unadjusted model	Reference	2.29 (2.12‒2.46)	Reference	1.90 (1.78‒2.02)	Reference	2.49 (2.35‒2.65)	< 0.0001
Model 1	Reference	2.24 (2.08‒2.42)	Reference	1.83 (1.71‒1.94)	Reference	2.45 (2.31‒2.60)	< 0.0001
Model 2	Reference	1.85 (1.71‒1.99)	Reference	1.55 (1.45‒1.65)	Reference	2.07 (1.94‒2.20)	< 0.0001
Model 3	–	–	Reference	1.21 (1.12‒1.30)	Reference	1.76 (1.64‒1.88)	< 0.0001

Model 1: adjusted for sex, age, smoking habits, alcohol consumption, physical activities, family history of diabetes, antihypertensives, and lipid-lowering drugs

Model 2: further adjusted for *log*(TG/HDL-C) (continuous), SBP (continuous), and *log*hsCRP (continuous, entire cohort only)

Model 3: additionally adjusted for BMI (continuous) on the basis of Model 2

The incidence rate is per 1000 person-years

INTm: multiplicative interaction, others as Table 1

*P*-INTm: BMI subgroups*hsCRP subgroup (< 2, ≥ 2 mg/L) = 0.4978; WC subgroups*hsCRP subgroup (< 2, ≥ 2 mg/L) = 0.8065; Fatty liver subgroup*hsCRP subgroup (< 2, ≥ 2 mg/L) = 0.8043

**Table 3:** Additive effect of increased adiposity and inflammation in incident type 2 diabetes

Main effects–hazard ratios	Overweight and obesity (BMI)	Central obesity (Waist circumference)	Fatty liver
Model 1			
Increased adiposity	2.15 (2.06‒2.25)	1.78 (1.71‒1.85)	2.47 (2.37‒2.57)
Increased inflammation	1.16 (1.07‒1.25)	1.16 (1.09‒1.23)	1.15 (1.09‒1.22)
Joint effect	2.61 (2.48‒2.75)	2.10 (2.00‒2.20)	2.84 (2.70‒2.97)
RERI	0.30 (0.17‒0.43)	0.16 (0.05‒0.27)	0.22 (0.08‒0.36)
AP	0.12 (0.07‒0.16)	0.08 (0.03‒0.13)	0.08 (0.03‒0.13)
S	1.23 (1.12‒1.35)	1.17 (1.05‒1.31)	1.14 (1.04‒1.23)
Attributable proportion, (%)			
Increased adiposity	71.43	70.91	79.89
Increased inflammation	9.94	14.55	8.15
Additive interaction	18.63	14.54	11.96
Model 2			
Increased adiposity	1.77 (1.69‒1.85)	1.67 (1.61‒1.74)	2.03 (1.95‒2.12)
Elevated inflammation	1.14 (1.06‒1.23)	1.15 (1.08‒1.21)	1.12 (1.07‒1.18)
Joint effect	2.08 (1.97‒2.19)	1.94 (1.85‒2.03)	2.30 (2.19‒2.42)
RERI	0.17 (0.05‒0.28)	0.12 (0.02‒0.23)	0.15 (0.02‒0.27)
AP	0.08 (0.03‒0.14)	0.06 (0.01‒0.12)	0.06 (0.01‒0.11)
S	1.19 (1.05‒1.34)	1.15 (1.01‒1.30)	1.13 (1.02‒1.24)
Attributable proportion, (%)			
Increased adiposity	71.30	71.28	79.23
Increased inflammation	12.96	15.96	9.23
Additive interaction	15.74	12.76	11.54

Model 1: adjusted for sex, age, smoking habits, alcohol consumption, physical activities, family history of diabetes, antihypertensives, lipid-lowering drugs. Model 2: further adjusted for *log*(TG/HDL-C) (continuous) and SBP (continuous)

*AP* attributable proportion due to interaction, *RERI* relative excess risk due to interaction, *S* synergy index; others are as in Table 1

We additionally examined the hsCRP-diabetes risks when stratifying by cutoffs (1, 3 mg/L). Compared to those with hsCRP < 1 mg/L, the adjusted risks were 1.22 (95% CI 1.17‒1.27) and 1.23 (95% CI 1.18‒1.29), respectively, for 1 ≤ hsCRP < 3 and hsCRP ≥ 3 mg/L. Those with hsCRP ≥ 3 mg/L had significant diabetic risks (1.15, 95% CI 1.11‒1.20) when compared to those without. These associations remained significant after additional adjustment for BMI. When repeating the analysis by defining elevated inflammation with hsCRP levels ≥ 3 mg/L, similar results regarding the diabetic risks and significant additive interaction upon BMI-inflammation co-exposure were obtained (Additional file 1: Tables S9–S13). Consistent results with the main results were documented in the sensitivity analyses when excluding participants with baseline CVD, with suspected infection (hsCRP ≥ 10 mg/L), with diabetes onset within the first follow-up survey, or with missing values on the study covariates (Additional file 1: Tables S14–S21).

## Discussion

In a large-scale, real-life, prospective cohort, we found that both elevated inflammation and adiposity (assessed by BMI, waist circumference, and fatty liver) were independently and jointly associated with incident T2D. For BMI-indicated adiposity, the contributed proportions of the joint association were 12.96% for elevated inflammation alone, 71.30% for BMI-indicated excess body weight, and 15.74% for their additive interaction. The presence of fatty liver alone accounted for 79.23% of incident diabetes, while the contributed proportion of the interaction between elevated inflammation and fatty liver was 11.54%.

Adiposity heightens the risk of incident diabetes [4]. Our study, for the first time, provided epidemiological evidence on the significant modified effect of inflammation on excess weight-associated diabetes risks and highlighted the supra-additive interaction between adiposity and elevated inflammation in diabetogenesis. Notably, if concurrent increased adiposity and inflammation were present, this would result in an additional 11.54% ~ 15.74% of T2D cases. From a public health standpoint, because a predominant proportion (71% for excess weight to 79% for fatty liver) of the joint effect could be attributed to adiposity, our findings underscore the importance of maintaining an ideal body weight for T2D prevention. These findings are consistent with previous findings from other high-quality well-designed interventional studies [28, 29] as well as with current public health recommendations [4, 22, 30]. Additionally, although the inflammation-diabetes association was not conclusive that adiposity may confound the correlation [11–14], our results showed that in mainland China, elevated inflammation was associated with incident type 2 diabetes, independent of body weight. This is consistent with the results from a nationwide study (CHARLS) in China [10]. The augmented hsCRP-diabetes association among overweight/obese individuals in the CHARLS study supported our findings. Coupled with, results from the UK Biobank revealing that participants exposed to air pollution had more pronounced risks of incident diabetes among obese individuals than non-obese individuals [31] also provided similar conclusions to our study, as air pollution potentially reinforces a proinflammatory milieu [32]. Apart from the genetic predisposition, the modified effect of elevated inflammation should also partly explain the risk heterogeneities for incident diabetes upon accumulation of body fat.

Moreover, our results may provide epidemiological insight into the potential of anti-inflammatory pharmacotherapy for T2D prevention. Currently, mainstream knowledge admires anti-inflammatory strategies for improving glucose homeostasis and β-cell function [6]. For example, agents neutralizing interleukin-1β (IL-1β) with a monoclonal antibody [33, 34] or IL-1 receptor antagonist [35] contributed to reductions in systemic inflammation and improvement in glucose metabolism. Nonetheless, in a secondary analysis of the diabetes endpoint in the CANVOS trial, blockade of IL-1β with canakinumab over a median period of 3.7 years did not reduce incident diabetes [36]. Although the probable involvement of other inflammatory pathways in diabetogenesis [e.g., the c-Jun N-terminal kinase (JNK) and nuclear factor-κB (NF-κB), tumor necrosis factor (TNF) pathways] and the highly selective study participants as well as potential lifestyle changes in the specific population were supposed to account for the overall inefficiency of canakinumab [6, 36] for diabetes prevention, our findings provided another explanation for the discouraging result. Notably, elevated TG levels (a 10% increase) were observed during canakinumab treatment [33]. According to the decomposition results in our study, adiposity predominated most of the risks of incident diabetes, and only approximately 10% of diabetic risks was ascribed to elevated inflammation. In this regard, the elevation of TG levels during treatment may heighten diabetic risks, which may surpass the antidiabetic effect of targeting inflammation. Moreover, most of the available antidiabetic agents potentiate an alleviation in overall inflammation [37]. Nonetheless, only three kinds of antidiabetic agents (glucagon-like peptide analogues [38], metformin [28, 39] and sodium-glucose cotransporter-2 inhibitors [40] have proven benefits in preventing T2D and its complications. In contrast to other kinds of widely used antidiabetic agents that frequently lead to weight gain during treatment, e.g., insulin, sulfonylurea or thiazolidinediones [37], these kinds of drugs potentiate the dual merits of weight loss and inflammation reduction. In light of our findings here, approaches simultaneously targeting fatness and inflammation should achieve greater benefits than targeting each individual risk factor. More diabetes-focused, prospective, interventional studies are warranted to investigate the effect of anti-inflammatory agents on body weight change and the potential for T2D prevention.

Notably, in the context of public health, the additivity of effect commonly reflects a causal or biological relationship [27]. Indeed, converged effort has been devoted to understanding the pathogenesis of obesity-derived systemic inflammation and obesity-related diabetogenesis. Type 2 diabetes manifests when pancreatic β-cells fail to adapt to the increased insulin demand caused by insulin resistance. Nutrient overload is an obvious feature of overweight/obesity and can drive the release of reactive oxygen species, resulting in oxidative stress [41] and increased influx of promote endoplasmic reticulum stress and contribute to β-cell lipotoxicity [42]. It is noteworthy that each of the cellular stress-related pathophysiology is likely to either induce an inflammatory response or to be exacerbated by or associated with inflammation [6, 43]. Both innate and adaptive immunity participate in the pathophysiology, mechanically involving proinflammatory pathways, e.g., stress-activated JNK and NF-κB [6], and/or macrophage-inflammasome-IL-1β-related signaling [44]. These processes consequently lead to islet cell apoptosis, not only impairing insulin secretion but also affecting other key tissues (e.g., skeletal muscle or the liver) involved in the regulation of glucose metabolism [45]. The potential crosstalk between immunity and metabolism provides a biological basis for the significant supra-additive interactions found in our study.

### Clinical implications

The globe is experiencing a dual epidemic of obesity and T2D, and the situation is worsening. Projections estimate a sixfold increase in the number of obese adults and an increase in diabetes prevalence to 642 million by 2040. China has the world’s largest diabetes and prediabetes epidemics [2]. The rapid changes in urbanization and lifestyles have thus likely resulted in sustained increases in overall adiposity and diabetes prevalence in China. Lifestyle management targeting excess weight is the primary approach for T2D prevention. Adherence to regular physical activity and a calorie-reducing diet can significantly reduce overweight and obesity in prevalence and overall inflammatory levels [46], thereby contributing to T2D prevention. A recent study focusing on obese participants at highest risk of developing diabetes showed a 39% absolute risk reduction of developing diabetes with intensive lifestyle intervention [39]. Additionally, anti-inflammatory pharmacotherapy [4] should act as an important adjunct to lifestyle changes for the prevention of diabetes. Currently available antidiabetic agents, including glucagon-like peptide analogues [38], metformin [28, 39] and SGLT2 inhibitors [40], should excel themselves in diabetes prevention due to their benefits in both weight loss and inflammation reduction. Furthermore, our results support the beneficial potential of anti-inflammatory agents targeting specific inflammatory pathways for preventing diabetes, especially among those with adiposity. The combined use of these agents with other weight loss drugs is merited if these drugs have no significant reduction in body weight.

The strengths of the current study are the comprehensive investigation of the association between adiposity phenotypes (including general, central and fatty liver) and incident T2D and specifically across different levels of hsCRP, considering their interaction on both multiplicative and additive scales. Additionally, this is the first study to qualify the relative contributions of adiposity and elevated inflammation to incident diabetes and provide epidemiological evidence regarding the diabetes-preventive potential of anti-inflammatory treatment. Other merits of this study included the large study sample size, the prospectively designed and real-world cohort and the high quality of the data processing and collection.

The study also has certain limitations. First, the study was conducted primarily among the Han Chinese population in northern China, which limits the generalizability of the findings to the entire country and/or other ethnic groups. However, the relative homogeneity in the diet patterns, occupational and environmental exposures among the community-dwelling adults should reduce potential confounding. Second, the occupation-specific cohort wherein a great proportion of participants are coal mining workers may have also limited the generalizability of the findings to other occupations. Nonetheless, data from the nationwide cohort in China (CHARLS) also documented a stronger association between hsCRP and T2D onset among overweight individuals [10], consistent with our findings. Third, oral glucose tolerance testing or hemoglobin A1c measurement was not available in the study cohort. The diagnosis of T2D was only based on a single measurement of FBG, which may inevitably lead to misestimation of the incidence of T2D. Fourth, although we have tried to comprehensively assess adiposity by measures of BMI, waist circumference, and ultrasonography-measured fatty liver, it takes neither the muscle and fat mass relation nor directly measured fat distribution into account. Nonetheless, all anthropometrics were performed by well-trained experts rather than in a self-reported manner, which would ensure the reliability of the results. Fifth, we failed to distinguish type 1 from T2D in the study. Although T2D predominates > 95% of all cases of diabetes in the Chinese population and the greater age of the study participants compared to the usual type 1 diabetes onset age may have minimized the bias, some degree of misclassification is therefore inevitable.

## Conclusions

In a real-life, prospective cohort in China, elevations in both weight and inflammation were associated with a higher risk of type 2 diabetes, and the joint effect was higher than the addition of the risks associated with each individual factor. Our findings suggest that most cases of type 2 diabetes could be prevented by adherence to a weight-loss intervention, and simultaneously targeting inflammation and adiposity would achieve greater benefits than targeting each individual point alone. Further studies are warranted to evaluate the diabetes-preventive potential of anti-inflammatory therapy by considering their combined effect on adiposity.

### Supplementary Information

**Additional file1: Figure S1.** Flowchart of the study participants. **Table S1**. Baseline characteristics of raw data. **Table S2.** HsCRP-associated type 2 diabetes risks in the entire cohort (14278/82172). **Table S3.** BMI(China)-associated risk of incident type 2 diabetes in the entire cohort and stratified by hsCRP strata (<2, ≥2 mg/L). **Table S4.** Fatty liver-associated risk of incident type 2 diabetes in the entire cohort and stratified by hsCRP strata (<2, ≥2 mg/L). **Table S5.** The risks of incident type 2 diabetes upon adiposity indices with adjustment for the product term of multiplicative interaction with hsCRP (<2, ≥2 mg/L). **Table S6.** The risks of incident type 2 diabetes upon co-exposure to increased BMI and hsCRP levels. **Table S7.** The risks of incident type 2 diabetes upon co-exposure to central obesity (waist circumference) and elevated hsCRP levels. **Table S8.** The risks of incident type 2 diabetes upon co-exposure to fatty liver and elevated hsCRP levels. **Table S9.** HsCRP-associated risk of incident type 2 diabetes in the entire cohort and stratified by hsCRP strata (<1, 1~3, ≥3mg/L). **Table S10.** HsCRP-associated risk of incident type 2 diabetes in the entire cohort and stratified by hsCRP strata (<3, ≥3mg/L). **Table S11.** BMI-associated risk of incident type 2 diabetes in the entire cohort and stratified by hsCRP strata (<3, ≥3 mg/L). **Table S12.** Additive interaction of overweight/obesity and hsCRP≥3 mg/L. **Table S13.** The risks of incident type 2 diabetes upon co-exposure to BMI and hsCRP levels (<3, ≥3 mg/L). **Table S14.** Sensitivity analysis of the risks of incident type 2 diabetes upon co-exposure to BMI and hsCRP levels by excluding CVD (13685/79714). **Table S15.** Additive interaction of overweight/obesity and hsCRP≥2 mg/L by excluding individuals with pre-existing CVD (13685/79714). **Table S16.** Sensitivity analysis of risks of incident type 2 diabetes upon co-exposure to BMI and hsCRP levels by excluding suspected infection (13646/78992). **Table S17.** Additive interaction of overweight/obesity and hsCRP≥2 mg/L by excluding individuals with suspected infection (13646/78992). **Table S18.** Reverse analysis of the risks of incident type 2 diabetes upon co-exposure to BMI and hsCRP levels (<2, ≥2 mg/L) (10601/78495). **Table S19.** Additive interaction of overweight/obesity and hsCRP≥2 mg/L in the reverse analysis (10601/78495). **Table S20.** The risks of incident type 2 diabetes upon co-exposure to BMI and hsCRP levels (<2, ≥2 mg/L) on raw data. **Table S21.** Additive interaction of overweight/obesity and hsCRP≥2 mg/L on raw data.

## Data Availability

The datasets used and/or analyzed during the current study are available from the corresponding author on reasonable request.

## Abbreviations

AP: Attributable proportion due to interaction
BMI: Body mass index
CVD: Cardiovascular disease
FBG: Fasting blood glucose
HDL-C: High-density lipoprotein cholesterol
hsCRP: High-sensitivity C-reactive protein
IL-1β: Interleukin-1β
INTa: Additive interaction
INTm: Multiplicative interaction
RERI: Relative excess risk due to interaction
SD: Standard deviation
TC: Total cholesterol
TG: Triglyceride
