# Supplementary material for: Combined effect of adiposity and elevated inflammation on incident type 2 diabetes: a prospective cohort study
Source: Cardiovasc Diabetol. 2023 Dec 20;22:351. doi: 10.1186/s12933-023-02067-0 (PMC10734163; doi:10.1186/s12933-023-02067-0)
Supplement: Supplementary file 1 — Additional file1: Figure S1. Flowchart of the study participants. Table S1. Baseline characteristics of raw data. Table S2. HsCRP-associated type 2 diabetes risks in the entire cohort (14278/82172). Table S3. BMI(China)-associated risk of incident type 2 diabetes in the entire cohort and stratified by hsCRP strata (<2, ≥2 mg/L). Table S4. Fatty liver-associated risk of incident type 2 diabetes in the entire cohort and stratified by hsCRP strata (<2, ≥2 mg/L). Table S5. The risks of incident type 2 diabetes upon adiposity indices with adjustment for the product term of multiplicative interaction with hsCRP (<2, ≥2 mg/L). Table S6. The risks of incident type 2 diabetes upon co-exposure to increased BMI and hsCRP levels. Table S7. The risks of incident type 2 diabetes upon co-exposure to central obesity (waist circumference) and elevated hsCRP levels. Table S8. The risks of incident type 2 diabetes upon co-exposure to fatty liver and elevated hsCRP levels. Table S9. HsCRP-associated risk of incident type 2 diabetes in the entire cohort and stratified by hsCRP strata (<1, 1~3, ≥3mg/L). Table S10. HsCRP-associated risk of incident type 2 diabetes in the entire cohort and stratified by hsCRP strata (<3, ≥3mg/L). Table S11. BMI-associated risk of incident type 2 diabetes in the entire cohort and stratified by hsCRP strata (<3, ≥3 mg/L). Table S12. Additive interaction of overweight/obesity and hsCRP≥3 mg/L. Table S13. The risks of incident type 2 diabetes upon co-exposure to BMI and hsCRP levels (<3, ≥3 mg/L). Table S14. Sensitivity analysis of the risks of incident type 2 diabetes upon co-exposure to BMI and hsCRP levels by excluding CVD (13685/79714). Table S15. Additive interaction of overweight/obesity and hsCRP≥2 mg/L by excluding individuals with pre-existing CVD (13685/79714). Table S16. Sensitivity analysis of risks of incident type 2 diabetes upon co-exposure to BMI and hsCRP levels by excluding suspected infection (13646/78992). Table S17. Additive interaction of [file 12933_2023_2067_MOESM1_ESM.docx]

Table of Contents

[eTable 1. Baseline characteristics of raw data 3](#_Toc150626657)

[eTable 2. HsCRP-associated type 2 diabetes risks in the entire cohort (14278/82172) 4](#_Toc150626658)

[eTable 3. BMI(China)-associated risk of incident type 2 diabetes in the entire cohort and stratified by hsCRP strata (<2, ≥2 mg/L) 5](#_Toc150626659)

[eTable 4. Fatty liver-associated risk of incident type 2 diabetes in the entire cohort and stratified by hsCRP strata (<2, ≥2 mg/L) 6](#_Toc150626660)

[eTable 5 The risks of incident type 2 diabetes upon adiposity indices with adjustment for the product term of multiplicative interaction with hsCRP (<2, ≥2 mg/L) 7](#_Toc150626661)

[eTable 6. The risks of incident type 2 diabetes upon co-exposure to increased BMI and hsCRP levels 8](#_Toc150626662)

[eTable 7. The risks of incident type 2 diabetes upon co-exposure to central obesity (waist circumference) and elevated hsCRP levels 9](#_Toc150626663)

[eTable 8. The risks of incident type 2 diabetes upon co-exposure to fatty liver and elevated hsCRP levels 10](#_Toc150626664)

[eTable 9. HsCRP-associated risk of incident type 2 diabetes in the entire cohort and stratified by hsCRP strata (<1, 1~3, ≥3mg/L) 11](#_Toc150626665)

[eTable 10. HsCRP-associated risk of incident type 2 diabetes in the entire cohort and stratified by hsCRP strata (<3, ≥3mg/L) 12](#_Toc150626666)

[eTable 11. BMI-associated risk of incident type 2 diabetes in the entire cohort and stratified by hsCRP strata (<3, ≥3 mg/L) 13](#_Toc150626667)

[eTable 12 Additive interaction of overweight/obesity and hsCRP≥3 mg/L 14](#_Toc150626668)

[eTable 13. The risks of incident type 2 diabetes upon co-exposure to BMI and hsCRP levels (<3, ≥3 mg/L) 15](#_Toc150626669)

[eTable 14. Sensitivity analysis of the risks of incident type 2 diabetes upon co-exposure to BMI and hsCRP levels by excluding CVD (13685/79714) 16](#_Toc150626670)

[eTable 15. Additive interaction of overweight/obesity and hsCRP≥2 mg/L by excluding individuals with pre-existing CVD (13685/79714) 17](#_Toc150626671)

[eTable 16. Sensitivity analysis of risks of incident type 2 diabetes upon co-exposure to BMI and hsCRP levels by excluding suspected infection (13646/78992) 18](#_Toc150626672)

[eTable 17. Additive interaction of overweight/obesity and hsCRP≥2 mg/L by excluding individuals with suspected infection (13646/78992) 19](#_Toc150626673)

[eTable 18. Reverse analysis of the risks of incident type 2 diabetes upon co-exposure to BMI and hsCRP levels (<2, ≥2 mg/L) (10601/78495) 20](#_Toc150626674)

[eTable 19. Additive interaction of overweight/obesity and hsCRP≥2 mg/L in the reverse analysis (10601/78495) 21](#_Toc150626675)

[eTable 20. The risks of incident type 2 diabetes upon co-exposure to BMI and hsCRP levels (<2, ≥2 mg/L) on raw data 22](#_Toc150626676)

[eTable 21. Additive interaction of overweight/obesity and hsCRP≥2 mg/L on raw data 23](#_Toc150626677)

[eFigure 1 Flowchart of the study participants. 24](#_Toc150626678)

# eTable 1. Baseline characteristics of raw data

|  | Total (n=82,172) | HsCRP<2 mg/L (n= 60,846) | HsCRP≥2 mg/L (n=21,326) | *P-*difference |
| --- | --- | --- | --- | --- |
| Age, mean (SD), years | 50.4±12.0 | 49.4±11.8 | 53.2±12.2 | <0.0001 |
| Male, No. (%) | 66047 (80.4) | 49241 (80.9) | 16806 (78.8) | <0.0001 |
| BMI, mean (SD), kg/m^2^ | 25.0±3.4 | 24.7±3.3 | 25.6±3.7 | <0.0001 |
| Waist circumference, mean (SD), cm | 86.6±9.7 | 85.6±9.4 | 89.3±10.1 | <0.0001 |
| Fatty liver degrees, No. (%) |  |  |  |  |
| No fatty liver | 57611 (70.1) | 44386 (73.0) | 13225 (62.0) | <0.0001 |
| Gentle fatty liver | 16548 (20.1) | 11378 (18.7) | 5170 (24.2) |  |
| Moderate and severe fatty liver | 8013 (9.8) | 5082 (8.3) | 2931 (13.7) |  |
| Fatty liver | 24561(29.9) | 16460 (27.0) | 8101 (38.0) |  |
| hsCRP, median (IQR), mg/L | 0.8 (0.3–2.1) | 0.5 (0.2–0.9) | 4.5 (2.8–7.8) | <0.0001 |
| SBP, mean (SD), mm Hg, MissingN=336 | 129.7±20.4 | 128.7±20.0 | 132.4±21.5 | <0.0001 |
| DBP, median (IQR), mm Hg, MissingN=374 | 80.0 (78.0–90.0) | 80.0 (77.0–90.0) | 80.7 (79.3–90.0) | <0.0001 |
| HDL-C, median (IQR), mmol/L | 1.50 (1.28–1.76) | 1.51 (1.28–1.77) | 1.49 (1.27–1.75) | 0.1001 |
| TC, mean (SD), mmol/L, MissingN=16 | 4.9±1.1 | 4.9±1.1 | 4.9±1.1 | 0.0026 |
| TG, median (IQR), mmol/L | 1.25 (0.88–1.88) | 1.22 (0.87–1.83) | 1.31(0.92–2.00) | <0.0001 |
| Family history of diabetes, No. (%) | 6823 (8.3) | 5230 (8.6) | 1593 (7.5) | <0.0001 |
| Education, No. (%), MissingN=45 |  |  |  | <0.0001 |
| Less than high school | 64957 (79.1) | 47905 (78.8) | 17052 (80.1) |  |
| High school and above | 17170 (20.9) | 12925 (21.2) | 4245 (19.9) |  |
| Drinking habits, No. (%), MissingN=82 |  |  |  |  |
| Current drinker | 31299 (38.1) | 24131 (39.7) | 7168 (33.7) | <0.0001 |
| Non-current drinker | 60791(61.9) | 36707 (60.3) | 14084(66.3) |  |
| Smoking habits, No. (%), MissingN=81 |  |  |  | <0.0001 |
| Never smoker | 48998 (59.7) | 37177 (61.1) | 11821 (55.6) |  |
| Ever smoker | 4452 (5.4) | 3170 (5.2) | 1282 (6.0) |  |
| Current smoker | 28641 (34.9) | 20464 (33.7) | 8177 (38.4) |  |
| Physical activities, No. (%), MissingN=362 |  |  |  | <0.0001 |
| Low | 7402 (9.1) | 5680 (9.4) | 1722 (8.2) |  |
| Moderate | 61862 (75.6) | 45743 (75.3) | 16119 (76.6) |  |
| High | 12546 (15.3) | 9348 (15.4) | 3198 (15.2) |  |
| Hypertension, No. (%) | 33951 (41.3) | 23732 (39.0) | 10219 (47.9) | <0.0001 |
| CVD, No. (%) | 2458 (3.0) | 1588 (2.6) | 870 (4.1) | <0.0001 |
| Dyslipidemia, No. (%) | 23976 (29.2) | 17188 (28.3) | 6788 (31.8) | <0.0001 |
| Medication use, No. (%) |  |  |  |  |
| Antihypertensives | 2238 (2.7) | 1426 (2.3) | 812 (3.8) | <0.0001 |
| Statin | 175 (0.2) | 117 (0.2) | 58 (0.3) | <0.0001 |
| Fibrate | 64 (0.08) | 38 (0.06) | 26 (0.12) | 0.0299 |

Abbreviations: BMI: body mass index; hsCRP: high-sensitivity C-reactive protein; HDL-C: high-density lipoprotein cholesterol; TC: total cholesterol; TG: triglyceride; SBP: systolic blood pressure; CVD: cardiovascular diseases. eTable 2. HsCRP-associated type 2 diabetes risks in the entire cohort (14278/82172)

|  | HsCRP, HRs (95% CIs) | | *P*-trend | Per SD |
| --- | --- | --- | --- | --- |
|  | hsCRP<2 mg/L | hsCRP≥2 mg/L |  |  |
| Event/Total | 9854/60846 | 4424/21326 |  |  |
| Incidence rate | 16.73 | 22.36 |  |  |
| Crude model | Reference | 1.33 (1.28‒1.38) | <0.0001 | 1.19 (1.17‒1.21) |
| Model 1 | Reference | 1.27 (1.22‒1.31) | <0.0001 | 1.16 (1.14‒1.18) |
| Model 2 | Reference | 1.20 (1.16‒1.25) | <0.0001 | 1.12 (1.10‒1.14) |
| Model 3 | Reference | 1.12 (1.08‒1.16) | <0.0001 | 1.06 (1.04‒1.08) |

Model 1: adjusted for sex, age (continuous), smoking habits, alcohol consumption, physical activities, family history of diabetes, antihypertensives and lipid-lowering drugs.

Model 2: further adjusted for *log*(TG/HDL-C) (continuous) and SBP (continuous) on the basis of Model 1.

Model 3: further adjusted for BMI (continuous) on the basis of Model 2.

Per SD: risks of incident type 2 diabetes per-SD increase in *log*hsCRP (0.6639).

The incidence rate is per 1,000 person-years.

# eTable 3. BMI(China)-associated risk of incident type 2 diabetes in the entire cohort and stratified by hsCRP strata (<2, ≥2 mg/L)

|  | BMI, HRs (95% CIs) | | | *P*-trend | Per SD |
| --- | --- | --- | --- | --- | --- |
|  | BMI<24 kg/m^2^ | 24≤BMI<28 kg/m^2^ | BMI≥28 kg/m^2^ |  |  |
| Entire population | | | | | |
| Event/Total | 3443/33220 | 6852/34273 | 4253/14679 |  |  |
| Incidence rate | 10.37 | 20.27 | 32.67 |  |  |
| Unadjusted model | Reference | 1.95 (1.87‒2.03) | 3.13 (2.99‒3.27) | <0.0001 | 1.51 (1.49‒1.54) |
| Model 1 | Reference | 1.88 (1.81‒1.96) | 3.03 (2.89‒3.17) | <0.0001 | 1.51 (1.49‒1.53) |
| Model 2 | Reference | 1.58 (1.52‒1.65) | 2.26 (2.15‒2.37) | <0.0001 | 1.37 (1.35‒1.39) |
| hsCRP<2 mg/L (9854/60846) | | | | | |
| Event/Total | 2573/25937 | 4606/25283 | 2675/9626 |  |  |
| Incidence rate | 9.87 | 19.04 | 30.92 |  |  |
| Unadjusted model | Reference | 1.93 (1.84‒2.02) | 3.12 (2.95‒3.29) | <0.0001 | 1.54 (1.51‒1.57) |
| Model 1 | Reference | 1.85 (1.76‒1.94) | 3.00 (2.84‒3.17) | <0.0001 | 1.52 (1.50‒1.55) |
| Model 2 | Reference | 1.57 (1.50‒1.65) | 2.29 (2.16‒2.42) | <0.0001 | 1.39 (1.36‒1.42) |
| hsCRP≥2 mg/L (4424/21326) | | | | | |
| Event/Total | 870/7283 | 1976/8990 | 1578/5053 |  |  |
| Incidence rate | 12.21 | 23.84 | 36.13 |  |  |
| Unadjusted model | Reference | 1.94 (1.80‒2.11) | 2.93(2.70‒3.18) | <0.0001 | 1.44 (1.40‒1.47) |
| Model 1 | Reference | 1.91 (1.76‒2.07) | 2.88 (2.65‒3.13) | <0.0001 | 1.44 (1.40‒1.47) |
| Model 2 | Reference | 1.64 (1.52‒1.78) | 2.27 (2.08‒2.48) | <0.0001 | 1.34 (1.30‒1.37) |
| *P*-INTm: BMI subgroup*hsCRP subgroup (<2, ≥2 mg/L) = 0.5497. | | | | | |

Model 1: adjusted for sex, age, smoking habits, alcohol consumption, physical activities, family history of diabetes, antihypertensives, and lipid-lowering drugs.

Model 2: further adjusted for *log*(TG/HDL-C) (continuous), SBP (continuous), and *log*hsCRP (continuous, entire cohort only).

Per SD: risk of incident type 2 diabetes per SD increase in BMI (3.44).

The incidence rate is per 1,000 person-years. Abbreviations: INTm: multiplicative interaction, others as eTable 1.

# eTable 4. Fatty liver-associated risk of incident type 2 diabetes in the entire cohort and stratified by hsCRP strata (<2, ≥2 mg/L)

|  | Fatty liver, HRs (95% CIs) | | |  |
| --- | --- | --- | --- | --- |
|  | Non-fatty liver | Gentle fatty liver | Moderate and severe fatty liver | *P*-trend |
| Entire population |  |  |  |  |
| Event/Total | 7169/57611 | 4226/16548 | 2883/8013 |  |
| Incidence rate | 12.60 | 28.17 | 42.56 |  |
| Unadjusted model | Reference | 2.23 (2.15‒2.32) | 3.36 (3.22‒3.51) | <0.0001 |
| Model 1 | Reference | 2.16 (2.08‒2.25) | 3.24 (3.10‒3.39) | <0.0001 |
| Model 2 | Reference | 1.82 (1.75‒1.90) | 2.48(1.37‒2.60) | <0.0001 |
| Model 3 | Reference | 1.59 (1.53‒1.66) | 2.00 (1.90‒2.10) | <0.0001 |
| HsCRP<2 mg/L (9854/60846) | | | | |
| Event/Total | 5307/44386 | 2801/11378 | 1746/5082 |  |
| Incidence rate | 12.03 | 26.97 | 39.73 |  |
| Unadjusted model | Reference | 2.24 (2.14‒2.34) | 3.30 (3.12‒3.48) | <0.0001 |
| Model 1 | Reference | 2.16 (2.07‒2.26) | 3.16 (3.00‒3.34) | <0.0001 |
| Model 2 | Reference | 1.84 (1.75‒1.93) | 2.44 (2.30‒2.59) | <0.0001 |
| Model 3 | Reference | 1.59 (1.51‒1.67) | 1.94 (1.83‒2.07) | <0.0001 |
| HsCRP≥2 mg/L (4424/21326) | | | | |
| Event/Total | 1862/13225 | 1425/5170 | 1137/2931 |  |
| Incidence rate | 14.56 | 30.86 | 47.77 |  |
| Unadjusted model | Reference | 2.11 (1.97‒2.26) | 3.24 (3.01‒3.49) | <0.0001 |
| Model 1 | Reference | 2.08 (1.94‒2.23) | 3.17 (2.94‒3.41) | <0.0001 |
| Model 2 | Reference | 1.82 (1.69‒1.95) | 2.58 (2.38‒2.79) | <0.0001 |
| Model 3 | Reference | 1.62 (1.50‒1.74) | 2.11 (1.94‒2.30) | <0.0001 |
| *P*-INTm: Fatty liver subgroup*HsCRP subgroup (<2, ≥2 mg/L) = 0.4493 (Model 2); =0.6661 (Model 3). | | | | |

Model 1: adjusted for sex, age, smoking habits, alcohol consumption, physical activities, family history of diabetes, antihypertensives, and lipid-lowering drugs.

Model 2: further adjusted for *log*(TG/HDL-C) (continuous), SBP (continuous) and *log*hsCRP (continuous, entire cohort only).

Model 3: further adjusted for BMI (continuous) on the basis of Model 2.

The incidence rate is per 1,000 person-years. Abbreviations: INTm: multiplicative interaction, others as eTable 1.

# eTable 5 The risks of incident type 2 diabetes upon adiposity indices with adjustment for the product term of multiplicative interaction with hsCRP (<2, ≥2 mg/L)

|  | BMI, HRs (95% CIs) | | *P*-INTm |
| --- | --- | --- | --- |
|  | BMI<24 kg/m^2^ | BMI≥24 kg/m^2^ |  |
| Event/Total | 3443/33220 | 10835/48952 |  |
| Incidence rates | 10.37 | 23.82 |  |
| Unadjusted model | Reference | 2.29 (2.20‒2.38) | - |
| Model 1 | Reference | 2.06 (1.83‒2.31) | 0.2921 |
| Model 2 | Reference | 1.72 (1.53‒1.93) | 0.4978 |
|  | Waist circumference, HRs (95% CIs) | | *P*-INTm |
|  | Non-central obesity | Central obesity |  |
| Event/Total | 6446/48935 | 7832/33237 |  |
| Incidence rates | 13.34 | 25.79 |  |
| Unadjusted model | Reference | 1.93 (1.87‒1.99) | - |
| Model 1 | Reference | 1.75 (1.58‒1.93) | 0.6098 |
| Model 2 | Reference | 1.49 (1.35‒1.65) | 0.8065 |
| Model 3 | Reference | 1.18 (1.07‒1.31) | 0.7306 |
|  | Fatty liver, HRs (95% CIs) | | *P*-INTm |
|  | Non-fatty liver | Fatty liver |  |
| Event/Total | 7169/57611 | 7109/24561 |  |
| Incidence rates | 12.60 | 32.64 |  |
| Unadjusted model | Reference | 2.58 (2.50‒2.67) | - |
| Model 1 | Reference | 2.46 (2.23‒2.72) | 0.9674 |
| Model 2 | Reference | 2.02 (1.83‒2.23) | 0.8366 |
| Model 3 | Reference | 1.73 (1.57‒1.92) | 0.8043 |

Model 1: adjusted for sex, age, smoking habits, alcohol consumption, physical activities, family history of diabetes, antihypertensives, lipid-lowering drugs, hsCRP (<2, ≥2 mg/L) and the interaction with hsCRP category (<2, ≥2 mg/L).

Model 2: further adjusted for log(TG/HDL-C) (continuous) and SBP (continuous).

Model 3: additionally adjusted for BMI (continuous) on the basis of Model 2.

The incidence rate is per 1,000 person-years.

# eTable 6. The risks of incident type 2 diabetes upon co-exposure to increased BMI and hsCRP levels

|  | BMI<24 kg/m^2^ & hsCRP<2 mg/L | BMI<24 kg/m^2^ & hsCRP≥2 mg/L | BMI≥24 kg/m^2^ & hsCRP<2 mg/L | BMI≥24 kg/m^2^ & hsCRP≥2 mg/L |
| --- | --- | --- | --- | --- |
| Event/Total | 2573/25937 | 870/7283 | 7281/34909 | 3554/14043 |
| Incident rate | 9.87 | 12.21 | 22.17 | 28.08 |
| Unadjusted model | Reference | 1.23 (1.14‒1.33) | 2.24 (2.14‒2.34) | 2.82 (2.68‒2.97) |
| Model 1 | Reference | 1.16 (1.07‒1.25) | 2.15 (2.06‒2.25) | 2.61 (2.48‒2.75) |
| Model 2 | Reference | 1.14 (1.06‒1.23) | 1.77 (1.69‒1.85) | 2.08 (2.97‒2.19) |
| *P*-INTm: hsCRP (<2, ≥2 mg/L) * BMI (<24, ≥24 kg/m^2^) = 0.4978 | | | | |

Model 1: adjusted for sex, age, smoking habits, alcohol consumption, physical activities, family history of diabetes, antihypertensives, and lipid-lowering drugs.

Model 2: further adjusted for *log*(TG/HDL-C) (continuous) and SBP (continuous) on the basis of Model 1.

The incidence rate is per 1,000 person-years.

Abbreviations: INTm: multiplicative interaction, others as eTable 1.

# eTable 7. The risks of incident type 2 diabetes upon co-exposure to central obesity (waist circumference) and elevated hsCRP levels

|  | Non-central obesity & hsCRP<2 mg/L | Non-central obesity & hsCRP≥2 mg/L | Central obesity & hsCRP<2 mg/L | Central obesity & hsCRP≥2 mg/L |
| --- | --- | --- | --- | --- |
| Event/Total | 4937/38859 | 1499/10076 | 4907/21987 | 2925/11250 |
| Incidence rate | 12.84 | 15.32 | 24.09 | 29.26 |
| Unadjusted model | Reference | 1.19 (1.12‒1.26) | 1.87 (1.80‒1.95) | 2.26 (2.16‒2.37) |
| Model 1 | Reference | 1.16 (1.09‒1.23) | 1.78 (1.71‒1.85) | 2.10 (2.00‒2.20) |
| Model 2 | Reference | 1.15 (1.08‒1.21) | 1.67 (1.61‒1.74) | 1.94 (1.85‒2.03) |
| Model 3 | Reference | 1.11 (1.05‒1.18) | 1.17 (1.12‒1.22) | 1.28 (1.21‒1.35) |
| *P*-INTm: WC subgroup * hsCRP subgroup (<2, ≥2 mg/L) = 0.8065 | | | | |

Model 1: adjusted for sex, age, smoking habits, alcohol consumption, physical activities, family history of diabetes, antihypertensives, and lipid-lowering drugs.

Model 2: further adjusted for *log*(TG/HDL-C) (continuous) and SBP (continuous) on the basis of Model 1.

Model 3: additionally adjusted for BMI (continuous).

The incidence rate is per 1,000 person-years.

Abbreviations: INTm: multiplicative interaction, others as eTable 1.

# eTable 8. The risks of incident type 2 diabetes upon co-exposure to fatty liver and elevated hsCRP levels

|  | Non-fatty liver & hsCRP<2 mg/L | Non-fatty liver & hsCRP≥2 mg/L | Fatty liver & hsCRP<2 mg/L | Fatty liver & hsCRP≥2 mg/L |
| --- | --- | --- | --- | --- |
| Event/Total | 5307/44386 | 1862/13225 | 4547/16460 | 2562/8101 |
| Incidence rate | 12.03 | 14.56 | 30.77 | 36.61 |
| Unadjusted model | Reference | 1.21 (1.14‒2.27) | 2.55 (2.45‒2.65) | 3.02 (2.88‒3.17) |
| Model 1 | Reference | 1.15 (1.09‒1.21) | 2.47 (2.37‒2.57) | 2.84 (2.70‒2.97) |
| Model 2 | Reference | 1.12 (1.07‒1.18) | 2.03 (1.95‒2.12) | 2.30 (2.19‒2.42) |
| Model 3 | Reference | 1.09 (1.03‒1.15) | 1.72 (1.64‒1.79) | 1.85 (1.76‒2.95) |
| *P*-INTm: Fatty liver subgroup*hsCRP subgroup (<2 , ≥2 mg/L)=0.8043 (Model 2);=0.8366 (Model 3) | | | | |

Model 1: adjusted for sex, age, smoking habits, alcohol consumption, physical activities, family history of diabetes, antihypertensives, and lipid-lowering drugs.

Model 2: further adjusted for *log*(TG/HDL-C) (continuous) and SBP (continuous) on the basis of Model 1.

The incidence rate is per 1,000 person-years.

Model 3: additionally adjusted for BMI (continuous) on the basis of Model 2.

# eTable 9. HsCRP-associated risk of incident type 2 diabetes in the entire cohort and stratified by hsCRP strata (<1, 1~3, ≥3mg/L)

|  | hsCRP, HRs (95% CIs) | | | *P*-trend |
| --- | --- | --- | --- | --- |
|  | hsCRP<1 mg/L | 1<hsCRP<3 mg/L | hsCRP≥3 mg/L |  |
| Event/Total | 7108/46631 | 4032/20170 | 3138/15371 |  |
| Incidence rate | 15.58 | 21.49 | 21.92 |  |
| Crude model | Reference | 1.37 (1.32‒1.43) | 1.40 (1.34‒1.46) | <0.0001 |
| Model 1 | Reference | 1.33 (1.28‒1.38) | 1.31 (1.26‒1.37) | <0.0001 |
| Model 2 | Reference | 1.22 (1.17‒1.27) | 1.23 (1.18‒1.29) | <0.0001 |
| Model 3 | Reference | 1.12 (1.07‒1.16) | 1.13 (1.08‒1.18) | <0.0001 |

Model 1: adjusted for sex, age (continuous), smoking habits, alcohol consumption, physical activities, family history of diabetes, antihypertensives, and lipid-lowering drugs.

Model 2: further adjusted for *log*(TG/HDL-C) (continuous) and SBP (continuous) on the basis of Model 1.

Model 3: further adjusted for BMI (continuous) on the basis of Model 2.

The incidence rate is per 1,000 person-years.

# eTable 10. HsCRP-associated risk of incident type 2 diabetes in the entire cohort and stratified by hsCRP strata (<3, ≥3mg/L)

|  | hsCRP, HRs (95% CIs) | | *P*-trend |
| --- | --- | --- | --- |
|  | hsCRP<3 mg/L | hsCRP>3 mg/L |  |
| Event/Total | 11140/66801 | 3138/15371 |  |
| Incidence rate | 17.31 | 21.92 |  |
| Crude model | Reference | 1.26 (1.21‒1.31) | <0.0001 |
| Model 1 | Reference | 1.19 (1.15‒1.24) | <0.0001 |
| Model 2 | Reference | 1.15 (1.11‒1.20) | <0.0001 |
| Model 3 | Reference | 1.08 (1.04‒1.13) | <0.0001 |

Model 1: adjusted for sex, age (continuous), smoking habits, alcohol consumption, physical activities, family history of diabetes, antihypertensives, and lipid-lowering drugs.

Model 2: further adjusted for *log*(TG/HDL-C) (continuous) and SBP (continuous) on the basis of Model 1.

Model 3: further adjusted for BMI (continuous) on the basis of Model 2.

The incidence rate is per 1,000 person-years.

# eTable 11. BMI-associated risk of incident type 2 diabetes in the entire cohort and stratified by hsCRP strata (<3, ≥3 mg/L)

|  | BMI, HRs (95% CIs) | | *P*-trend | Per SD |
| --- | --- | --- | --- | --- |
|  | BMI<24 kg/m^2^ | BMI≥24 kg/m^2^ |  |  |
| hsCRP<3 mg/L (11140/66801) | | | | |
| Event/Total | 2803/27808 | 8337/38993 |  |  |
| Incidence rate | 10.05 | 22.86 |  |  |
| Unadjusted model | Reference | 2.27 (2.17‒2.37) | <0.0001 | 1.53 (1.50‒1.56) |
| Model 1 | Reference | 2.17 (2.08‒2.27) | <0.0001 | 1.52 (1.49‒1.55) |
| Model 2 | Reference | 1.77 (1.69‒1.85) | <0.0001 | 1.39 (1.36‒1.42) |
| hsCRP≥3 mg/L (3138/15371) | | | | |
| Event/Total | 640/5412 | 2498/9959 |  |  |
| Incidence rate | 12.09 | 27.69 |  |  |
| Unadjusted model | Reference | 2.28 (2.09‒2.48) | <0.0001 | 1.44 (1.40‒1.48) |
| Model 1 | Reference | 2.24 (2.05‒2.45) | <0.0001 | 1.44 (1.40‒1.49) |
| Model 2 | Reference | 1.84 (1.68‒2.02) | <0.0001 | 1.34 (1.30‒1.39) |
| *P*-INTm: BMI subgroup*hsCRP subgroup (<3, ≥3 mg/L) = 0.6905. | | | | |

Model 1: adjusted for sex, age, smoking habits, alcohol consumption, physical activities, family history of diabetes, antihypertensives, and lipid-lowering drugs.

Model 2: further adjusted for *log*(TG/HDL-C) (continuous), SBP (continuous), and *log*hsCRP (continuous, entire cohort only).

Per SD: risk of incident type 2 diabetes per SD increase in BMI (3.44).

The incidence rate is per 1,000 person-years. Abbreviations: INTm: multiplicative interaction, others as eTable 1.

# eTable 12 Additive interaction of overweight/obesity and hsCRP≥3 mg/L

| Main effects ‒ hazard ratios | Model 1 | Model 2 |
| --- | --- | --- |
| Overweight and obesity | 2.18 (2.09‒2.27） | 2.01 (1.93‒2.10) |
| hsCRP≥3 mg/L | 1.11(1.02‒1.21) | 1.11 (1.02‒1.21) |
| Joint effect | 2.52 (2.39‒2.66) | 2.29 (2.17‒2.42) |
| RERI | 0.23 (0.09‒0.37) | 0.17 (0.03‒0.31) |
| AP | 0.09 (0.04‒0.15) | 0.07 (0.02‒0.13) |
| S | 1.18 (1.06‒1.31) | 1.15 (1.02‒1.29) |
| Attributable proportion, % | | |
| Metabolic disorders | 77.63 | 78.29 |
| hsCRP≥3 mg/L | 7.24 | 9.24 |
| Additive interaction | 15.13 | 13.17 |

Model 1: adjusted for sex, age, smoking habits, alcohol consumption, physical activities, family history of diabetes, antihypertensives, and lipid-lowering drugs.

Model 2: further adjusted for *log*(TG/HDL-C) (continuous) and SBP (continuous).

Abbreviations: RERI, relative excess risk due to interaction; AP, attributable proportion due to interaction; S, synergy index; others are as in eTable 1.

# eTable 13. The risks of incident type 2 diabetes upon co-exposure to BMI and hsCRP levels (<3, ≥3 mg/L)

|  | BMI<24 kg/m^2^ & hsCRP<3 mg/L | BMI<24 kg/m^2^ & hsCRP≥3 mg/L | BMI≥24 kg/m^2^ & hsCRP<3 mg/L | BMI≥24 kg/m^2^ & hsCRP≥3 mg/L |
| --- | --- | --- | --- | --- |
| Entire population |  |  |  |  |
| Event/Total | 2803/27808 | 640/5412 | 8337/38993 | 2498/9959 |
| Incident rate | 10.05 | 12.09 | 22.86 | 27.69 |
| Unadjusted model | Reference | 1.20 (1.10‒1.31) | 2.27 (2.17‒2.37) | 2.74 (2.59‒2.89) |
| Model 1 | Reference | 1.11 (1.02‒1.21) | 2.18 (2.09‒2.27) | 2.52 (2.39‒2.66) |
| Model 2 | Reference | 1.11 (1.02‒1.21) | 2.01 (1.93‒2.10) | 2.29 (2.17‒2.42) |
| *P*-INTm: hsCRP (<3, ≥3 mg/L) * BMI (<24, ≥24 kg/m^2^) = 0.6905 | | | | |

Model 1: adjusted for sex, age, smoking habits, alcohol consumption, physical activities, family history of diabetes, antihypertensives, and lipid-lowering drugs.

Model 2: further adjusted for *log*(TG/HDL-C) (continuous) and SBP (continuous) on the basis of Model 1.

# eTable 14. Sensitivity analysis of the risks of incident type 2 diabetes upon co-exposure to BMI and hsCRP levels by excluding CVD (13685/79714)

|  | BMI<24 kg/m^2^ & hsCRP<2 mg/L | BMI<24 kg/m^2^ & hsCRP≥2 mg/L | BMI≥24 kg/m^2^ & hsCRP<2 mg/L | BMI≥24 kg/m^2^ & hsCRP≥2 mg/L |
| --- | --- | --- | --- | --- |
| Event/Total | 2501/25408 | 829/7068 | 6978/33850 | 3377/13388 |
| Incident rate | 9.76 | 11.93 | 21.82 | 27.84 |
| Unadjusted model | Reference | 1.22 (1.13‒1.32) | 2.23 (2.13‒2.33) | 2.83 (2.69‒2.98) |
| Model 1 | Reference | 1.15 (1.06‒1.24) | 2.15 (2.05‒2.25) | 2.64 (2.50‒2.77) |
| Model 2 | Reference | 1.13 (1.04‒1.22) | 1.75(1.67‒1.84) | 2.09 (1.98‒2.20) |
| *P*-INTm: hsCRP (<2, ≥2 mg/l) * BMI (<24, ≥24 kg/m^2^) = 0.2367 | | | | |

Model 1: adjusted for sex, age, smoking habits, alcohol consumption, physical activities, family history of diabetes, antihypertensives, and lipid-lowering drugs.

Model 2: further adjusted for *log*(TG/HDL-C) (continuous) and SBP (continuous) on the basis of Model 1.

The incidence rate is per 1,000 person-years. Abbreviations: INTm: multiplicative interaction, others as eTable 1.

# eTable 15. Additive interaction of overweight/obesity and hsCRP≥2 mg/L by excluding individuals with pre-existing CVD (13685/79714)

| Main effects ‒ hazard ratios | Model 1 | Model 2 |
| --- | --- | --- |
| Overweight and obesity | 2.15 (2.05‒2.25) | 1.75 (1.67‒1.84) |
| hsCRP≥2 mg/L | 1.15 (1.06‒1.24) | 1.13 (1.04‒1.22) |
| Joint effect | 2.64 (2.50‒2.78) | 2.09 (1.98‒2.20) |
| RERI | 0.34 (0.21‒0.48) | 0.21 (0.09‒0.32) |
| AP | 0.13 (0.08‒0.18) | 0.10 (0.04‒0.15) |
| S | 1.27 (1.15‒1.40) | 1.23 (1.08‒1.40) |

Model 1: adjusted for sex, age, smoking habits, alcohol consumption, physical activities, family history of diabetes, antihypertensives, and lipid-lowering drugs.

Model 2: further adjusted for *log*(TG/HDL-C) (continuous) and SBP (continuous) on the basis of Model 1.

Abbreviations: RERI, relative excess risk due to interaction; AP, attributable proportion due to interaction; S, synergy index; others are as in eTable 1.

# eTable 16. Sensitivity analysis of risks of incident type 2 diabetes upon co-exposure to BMI and hsCRP levels by excluding suspected infection (13646/78992)

|  | BMI<24 kg/m^2^ & hsCRP<2 mg/L | BMI<24 kg/m^2^ & hsCRP≥2 mg/L | BMI≥24 kg/m^2^ & hsCRP<2 mg/L | BMI≥24 kg/m^2^ & hsCRP≥2 mg/L |
| --- | --- | --- | --- | --- |
| Event/Total | 2573/25937 | 751/6150 | 7281/34909 | 3041/11996 |
| Incident rate | 9.87 | 12.47 | 22.17 | 28.11 |
| Unadjusted model | Reference | 1.26 (1.16‒1.36) | 2.24 (2.14‒2.34) | 2.83 (2.68‒2.98) |
| Model 1 | Reference | 1.18 (1.09‒1.28) | 2.15 (2.06‒2.25) | 2.62 (2.48‒2.76) |
| Model 2 | Reference | 1.16 (1.07‒1.26） | 1.76 (1.68‒1.85) | 2.06 (1.96‒2.18) |
| *P*-INTm: hsCRP (<2, ≥2 mg/L) * BMI (<24, ≥24 kg/m^2^) = 0.7813 | | | | |

Model 1: adjusted for sex, age, smoking habits, alcohol consumption, physical activities, family history of diabetes, antihypertensives, and lipid-lowering drugs.

Model 2: further adjusted for *log*(TG/HDL-C) (continuous) and SBP (continuous) on the basis of Model 1.

The incidence rate is per 1,000 person-years. Abbreviations: INTm: multiplicative interaction, others as eTable 1.

# eTable 17. Additive interaction of overweight/obesity and hsCRP≥2 mg/L by excluding individuals with suspected infection (13646/78992)

| Main effects ‒ hazard ratios | Model 1 | Model 2 |
| --- | --- | --- |
| Overweight and obesity | 2.15 (2.06‒2.25) | 1.76 (1.68‒1.85) |
| hsCRP≥2 mg/L | 1.18 (1.09‒1.28) | 1.16 (1.07‒1.26) |
| Joint effect | 2.62 (2.48‒2.76) | 2.06 (1.96‒2.18) |
| RERI | 0.28 (0.14‒0.42) | 0.15 (0.02‒0.27) |
| AP | 0.11 (0.06‒0.16) | 0.07 (0.01‒0.13) |
| S | 1.21 (1.10‒1.34) | 1.16 (1.02‒1.32) |

Model 1: adjusted for sex, age, smoking habits, alcohol consumption, physical activities, family history of diabetes, antihypertensives, and lipid-lowering drugs.

Model 2: further adjusted for *log*(TG/HDL-C) (continuous) and SBP (continuous) on the basis of Model 1.

Abbreviations: RERI, relative excess risk due to interaction; AP, attributable proportion due to interaction; S, synergy index; others are as in eTable 1.

# eTable 18. Reverse analysis of the risks of incident type 2 diabetes upon co-exposure to BMI and hsCRP levels (<2, ≥2 mg/L) (10601/78495)

|  | BMI<24 kg/m^2^ & hsCRP<2 mg/L | BMI<24 kg/m^2^ & hsCRP≥2 mg/L | BMI≥24 kg/m^2^ & hsCRP<2 mg/L | BMI≥24 kg/m^2^ & hsCRP≥2 mg/L |
| --- | --- | --- | --- | --- |
| Event/total | 1936/25300 | 627/7040 | 5472/33100 | 2566/13055 |
| Incidence rate | 7.45 | 8.83 | 16.76 | 20.45 |
| Unadjusted model | Reference | 1.19 (1.09‒1.30) | 2.27 (2.16‒2.39) | 2.78 (2.62‒2.95) |
| Model 1 | Reference | 1.14 (1.04‒1.25) | 2.20 (2.09‒2.32) | 2.65 (2.49‒2.81) |
| Model 2 | Reference | 1.13 (1.03‒1.23) | 1.81 (1.72‒1.91) | 2.10 (1.98‒2.24) |
| *P*-INTm: hsCRP (<2, ≥2 mg/L) * BMI (<24, ≥24 kg/m^2^) = 0.4357 | | | | |

Model 1: adjusted for sex, age, smoking habits, alcohol consumption, physical activities, family history of diabetes, antihypertensives, and lipid-lowering drugs.

Model 2: further adjusted for *log*(TG/HDL-C) (continuous) and SBP (continuous) on the basis of Model 1.

The incidence rate is per 1,000 person-years. Abbreviations: INTm: multiplicative interaction, others as eTable 1.

# eTable 19. Additive interaction of overweight/obesity and hsCRP≥2 mg/L in the reverse analysis (10601/78495)

| Main effects ‒ hazard ratios | Model 1 | Model 2 |
| --- | --- | --- |
| Overweight and obesity | 2.20 (2.09‒2.32) | 1.81 (1.72‒1.91) |
| hsCRP≥2 mg/L | 1.14 (1.04‒1.25) | 1.13 (1.03‒1.23) |
| Joint effect | 2.65 (2.49‒2.81) | 2.10 (1.98‒2.24) |
| RERI | 0.30 (0.15‒0.45) | 0.17 (0.04‒0.30) |
| AP | 0.11 (0.06‒0.17) | 0.08 (0.02‒0.14) |
| S | 1.22 (1.10‒1.36) | 1.18 (1.03‒1.37) |

Model 1: adjusted for sex, age, smoking habits, alcohol consumption, physical activities, family history of diabetes, antihypertensives, and lipid-lowering drugs.

Model 2: further adjusted for *log*(TG/HDL-C) (continuous) and SBP (continuous) on the basis of Model 1.

Abbreviations: RERI, relative excess risk due to interaction; AP, attributable proportion due to interaction; S, synergy index; others are as in eTable 1.

# eTable 20. The risks of incident type 2 diabetes upon co-exposure to BMI and hsCRP levels (<2, ≥2 mg/L) on raw data

|  | BMI<24 kg/m^2^ & hsCRP<2 mg/L | BMI<24 kg/m^2^ & hsCRP≥2 mg/L | BMI≥24 kg/m^2^ & hsCRP<2 mg/L | BMI≥24 kg/m^2^ & hsCRP≥2 mg/L |
| --- | --- | --- | --- | --- |
| Unadjusted model | Reference | 1.23 (1.14‒1.33) | 2.24 (2.14‒2.34) | 2.82 (2.68‒2.97) |
| Model 1 | Reference | 1.17 (1.08‒1.26) | 2.15 (2.06‒2.25) | 2.62 (2.49‒2.76) |
| Model 2 | Reference | 1.15 (1.06‒1.24) | 1.76 (1.68‒1.85) | 2.08 (1.97‒2.19) |
| *P*-INTm: hsCRP (<2, ≥2 mg/L) * BMI (<24, ≥24 kg/m^2^) = 0.5081 | | | | |

Model 1: adjusted for sex, age, smoking habits, alcohol consumption, physical activities, family history of diabetes, antihypertensives, and lipid-lowering drugs.

Model 2: further adjusted for *log*(TG/HDL-C) (continuous) and SBP (continuous) on the basis of Model 1.

The incidence rate is per 1,000 person-years. Abbreviations: INTm: multiplicative interaction, others as eTable 1.

# eTable 21. Additive interaction of overweight/obesity and hsCRP≥2 mg/L on raw data

| Main effects ‒ hazard ratios | Model 1 | Model 2 |
| --- | --- | --- |
| Overweight and obesity | 2.15 (2.06‒2.25) | 1.76 (1.68‒1.85) |
| hsCRP≥2 mg/L | 1.17 (1.08‒1.26) | 1.15 (1.06‒1.24) |
| Joint effect | 2.62 (2.49‒2.76) | 2.08 (1.97‒2.19) |
| RERI | 0.30 (0.17‒0.43) | 0.17 (0.06‒0.29) |
| AP | 0.12 (0.07‒0.16) | 0.08 (0.03‒0.14) |
| S | 1.23 (1.13‒1.35) | 1.19 (1.05‒1.35) |

Model 1: adjusted for sex, age, smoking habits, alcohol consumption, physical activities, family history of diabetes, antihypertensives, and lipid-lowering drugs.

Model 2: further adjusted for *log*(TG/HDL-C) (continuous) and SBP (continuous) on the basis of Model 1.

Abbreviations: RERI, relative excess risk due to interaction; AP, attributable proportion due to interaction; S, synergy index; others are as in eTable 1.


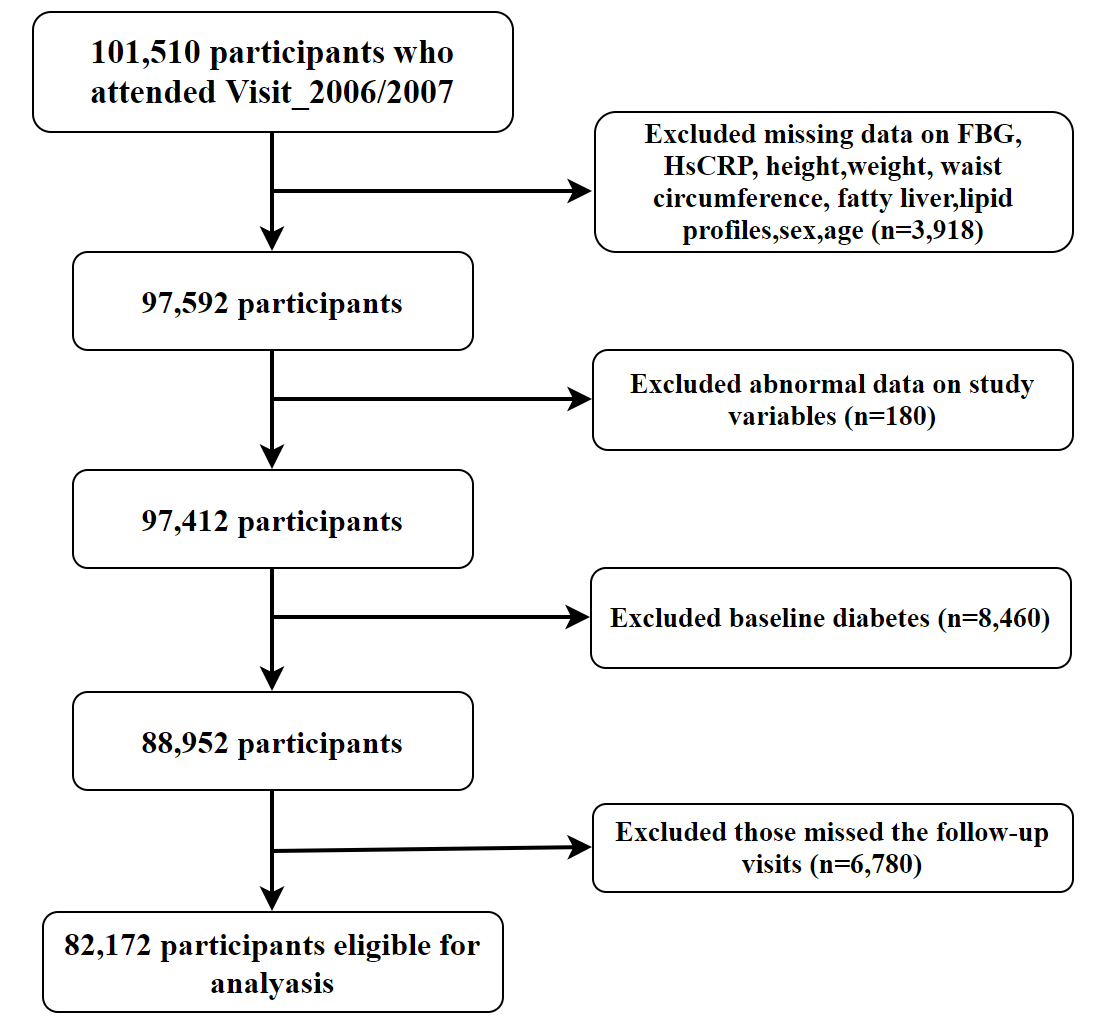


# eFigure 1 Flowchart of the study participants.
